# Gynæcology and Obstetrics

**Published:** 1906-02-03

**Authors:** 


### PROGRESS IN MEDICINE AND SURGERY.

### GYNÆCOLOGY AND OBSTETRICS.

**Eclampsia—its Pathology and Treatment.**—The pathology of eclampsia is obscure. It is a condition concerning which many theories have been advanced which are all really conjectural. Recent views, however, are no longer in favour of regarding the kidney as the main organ at fault, all important though it is in prophylaxis to secure efficient renal elimination. Suspicion now falls on a faulty metabolism of the liver rather than of the kidney, although in fatal cases of eclampsia parenchymatous changes are found in both organs. J. W. Byers,[4] of Belfast, in a recent article on the subject, shows in what a very large proportion of cases lesions of the liver are found. J. Whitridge Williams has figured in liver sections areas of red or white necrosis, secondary to thrombosis of the smaller portal vessels. Changes are, of course, found also in the kidneys and in the brain, but these, as well as those in the liver, are probably secondary rather than primary. Since the practical abandonment of the old view that eclampsia depended on a poisoning of the nerve-centres by certain by-products which a diseased kidney failed to eliminate, the toxæmic theory has become popular. This assumes the elaboration by the mother and fœtus of one or more toxins which, by accumulation, cause the post-mortem changes observed, and lead to the clinical manifestations; but, although there are many things to favour this view, no toxin has as yet been isolated, and eclampsia is still, in the words of Zweifel, "the disease of theories." Hastings Tweedy [5] believes that the eclamptic fit is due to a poison generated in the mother or the fœtus, and that this does not circulate in the blood as Byers and others think, but that it is probably deposited in the solid tissues, while Forbes Ross [6] points out that both views may be correct, for the early symptoms—headache, vomiting, dyspnœa, and albuminuria—suggest a circulating poison, while the later changes—parenchymatous degenerations in the liver and kidneys and affection of the nervous centres—indicate a close combination of the poison with the solid tissues. Many observers consider that the fœtus is one of the main factors causing the disease, and in some cases, as in a striking instance mentioned by Byers, albuminuria and eclamptic symptoms rapidly disappear if the fœtus dies. This is not always so, however. Of the eight cases of eclampsia recorded in the last report of the Rotunda Hospital [7] four of the infants were born in a state of maceration. Pre-existing renal disease is liable to be greatly aggravated by pregnancy, or, if arising during pregnancy it is acute and severe, as Boxall has pointed out. The albumin present is usually paraglobulin. Other important ætiological factors, the significance of which has not yet been decided, are the much greater frequency in primiparæ especially in association with mental anxiety, and in very young or elderly mothers, also in multiple pregnancy. It appears commoner also in certain years and in certain localities. Concerning the treatment of eclampsia as well as its pathology considerable diversity of opinion exists, at least as to the treatment of the actual fits. The symptoms of the pre-eclamptic state are albuminuria—although this may sometimes be absent, as Mrs. Scharlieb [8] has observed in India—diminished urinary and urea excretion, headache, giddiness, enfeebled vision, and severe epigastric pain. For these the indications are to spare and stimulate the eliminating organs, the skin, bowels, and especially the kidneys, indications best met by rest in bed, flannel clothing, warm baths, salines, and a non-nitrogenous diet consisting of milk and water in abundance, with moderate quantities of carbohydrates and vegetables. Thyroid extract has been recommended to increase renal efficiency. Opinions vary as to the best means of treating the convulsions when they occur, and as to the wisest obstetric practice, but there is evident an increasing disposition to discard chloroform in favour of morphia and to avoid inducing or unduly hastening labour. As Herbert Spencer [9] puts it, gentleness of treatment is advocated by those who have had most experience, and forced dilatation by Bossi's or other mechanical dilators is condemned by him, by Boxall, and others. So, also, is vaginal Cæsarian section, a severe and difficult procedure practised by Dührssen and recently advocated by Fry.[10] A study of 160 cases of eclampsia collected by Demelin [11] is instructive. Of this number 26 per cent. died after spontaneous delivery of the fœtus, 25 per cent. after delivery with forceps through a naturally dilated os, and 44 per cent. after delivery through an artificially dilated cervix. The high percentage mortality of the last class argues strongly against artificial dilatation. The special liability of the eclamptic patient to the absorption of poisons and also of strong antiseptics must be remembered. Where, however, the patient gets worse in spite of treatment, induction or hastening of labour may have to be undertaken. Morphia must be used cautiously but freely for the convulsions. Tweedy's method at the Rotunda is to inject half-a-grain, followed at intervals of two hours by quarter-grain doses until one grain or sometimes more has been given. Tweedy strongly deprecates giving anything by the mouth for fear of exciting œdema of the lungs, but advocates washing out the stomach and introducing hot saline water containing castor oil, salts, or croton oil. Action of the bowels is assisted also by hot saline injections as free purgation is of great importance. Saline infusions to dilute the poison are also very valuable but must not be pushed too far or they will lead to a return of the fits by altering the circulatory pressure and causing reabsorption of the toxins into the tissues according to Forbes Ross. A case of eclampsia in a Hindu woman, complicated by general septic peritonitis is reported by J. H. C. Leicester.[12] A large single splenic abscess was found after death, secondary it was thought to an antepartum infection of the uterus. Eclampsia associated with chorea is recorded by Gould and Howell.[13] In this case labour was induced at the fortieth week by artificial dilatation of the cervix with a Champetier de Ribes' bag. Both mother and child survived. A sudden appearance of albumin in the urine was rapidly followed by the first fit.

**Pernicious Vomiting of Pregnancy.**—A valuable paper on this subject is contributed by Whitridge Williams,[14] who records some important observations, which should be of value in prognosis and treatment. He considers that there is ground for dividing the serious vomiting of pregnancy into three classes: (1) reflex, (2) neurotic, and (3) toxæmic. Reflex vomiting may be due to something abnormal in the generative tract or ovum such as uterine displacements, especially retroflexions, endometritis, ovarian cysts, hydramnios, hydatidiform mole, multiple pregnancy, etc. Abnormal conditions of the cervix are probably not true factors in causing reflex vomiting. In all cases the urine should be carefully examined. Vomiting due to neurosis was first seriously considered by Kaltenbach in 1890. The fact that many cases are cured by suggestion or inefficient remedies shows that this is the explanation of certain of them; but many writers now ascribe far too many examples of vomiting to this cause. Organic lesions and toxæmia must always be eliminated. Certain cases, and perhaps these will be found an increasing group, belong to the toxæmias. Fischl, in 1884, appears to have been the first to suggest such an origin for the symptoms. He ascribed it to absorption of toxic material from the impacted large intestine. Later the occasional occurrence of pernicious vomiting and multiple neuritis was observed, pointing to a toxin circulating in the blood, and for the last ten years the toxæmic theory has been gaining ground. The secretion of the corpus luteum, or the ovary, intestinal absorption, hepato-toxæmia, and invasion of the maternal organism by fœtal elements (the cyncytio-toxin theory of Veit, Belim, and others) have been suggested as possible causes of the toxin; while some observers, as Champetier de Ribes and others, maintain the identity of the toxin with that of eclampsia on the one hand, and acute yellow atrophy on the other. Jaundice sometimes occurs in the last stages of the disease, and as far back as 1879 Matthews Duncan suggested that some of the fatal cases of hyperemesis were really examples of acute yellow atrophy of the liver. In a fatal case of his own in 1903, in which vomiting persisted in spite of induction of abortion at the third month, Williams found at the necropsy lesions characteristic of acute yellow atrophy in the liver and kidneys—namely, fatty degeneration and necrosis of the central parts of the hepatic lobules and of the renal secreting substance. In all about ten cases of association of these two diseases have been recorded—a significant number when one recalls the rarity of acute yellow atrophy and of fatal hyperemesis. The nature of the toxin concerned is unknown. Presumably it is metabolic in origin, and connected with gestation; but whether elaborated by the mother or the fœtus, or both, is uncertain. That striking metabolic changes are present is shown by Williams, who has demonstrated a marked increase in the percentage of nitrogen put out as ammonia compared with the total nitrogen of the urine, so that the normal of 3 to 5 per cent. may even rise to 46 per cent. The cause of this increased ammonia coefficient is undecided. It may be that owing to the destruction of liver tissue nitrogen oxidation is interfered with, hence more escapes as ammonia instead of as the more highly oxidised urea. Or it may be that the excess of ammonia is designed to neutralise an excessive production of acid, the disease being due to an acid intoxication such as occurs in diabetes and phosphorus poisoning. The practical teaching of this very valuable observation is that abortion should be immediately induced in women suffering from pernicious vomiting if the ammonia coefficient of the discharged nitrogen is much increased; otherwise the toxæmia will progress to a fatal end, accompanied by lesions in the liver and kidneys. The determination of the proportion of ammonia is also valuable for prognosis as a favourable result without induction may be anticipated where it is not increased. With Stone and Ewing and others, who regard eclampsia as another manifestation of the same toxæmia, Williams strongly disagrees. He is supported by Schmorl when he points out that in eclampsia the necrotic lesions in the liver are very different to those in acute yellow atrophy or toxæmic vomiting, starting as they do in the portal system and involving the lobule from the periphery towards the centre, instead of starting in the centre and avoiding the portal system. The clinical manifestations of the two diseases are, of course, very different, and also the result of chemical examination of the urine. In eclampsia the total amount of nitrogen excreted is greatly reduced, the ammonia coefficient remaining practically normal; whereas, as has been shown, in toxæmic vomiting the total nitrogen keeping about normal the ammonia coefficient is greatly increased. A high ammonia output is therefore of bad prognosis in vomiting, but a favourable sign in eclampsia.

[4] Lancet., Sept. 9. [5] Ibid., Aug. 12, p. 459. [6] Ibid. [7] Dublin Jour. of Med. Sc., June. [8] Lancet, Aug. 12, p. 460. [9] Ibid. [10] Brit. Med. Jour., epitome, Dec. 9. [11] Ibid., Nov. 25. [12] Lancet, Sept. 16. [13] Ibid., Oct. 21. [14] Ibid., Oct. 21.

